# Supplementary material for: Small-RNA Sequencing Reveals Altered Skeletal Muscle microRNAs and snoRNAs Signatures in Weanling Male Offspring from Mouse Dams Fed a Low Protein Diet during Lactation
Source: Cells. 2021 May 11;10(5):1166. doi: 10.3390/cells10051166 (PMC8150574; doi:10.3390/cells10051166)
Supplement: Supplementary file 1 [file cells-10-01166-s001.zip › cells-1202102-supplementary.pdf]

Supplementary Materials

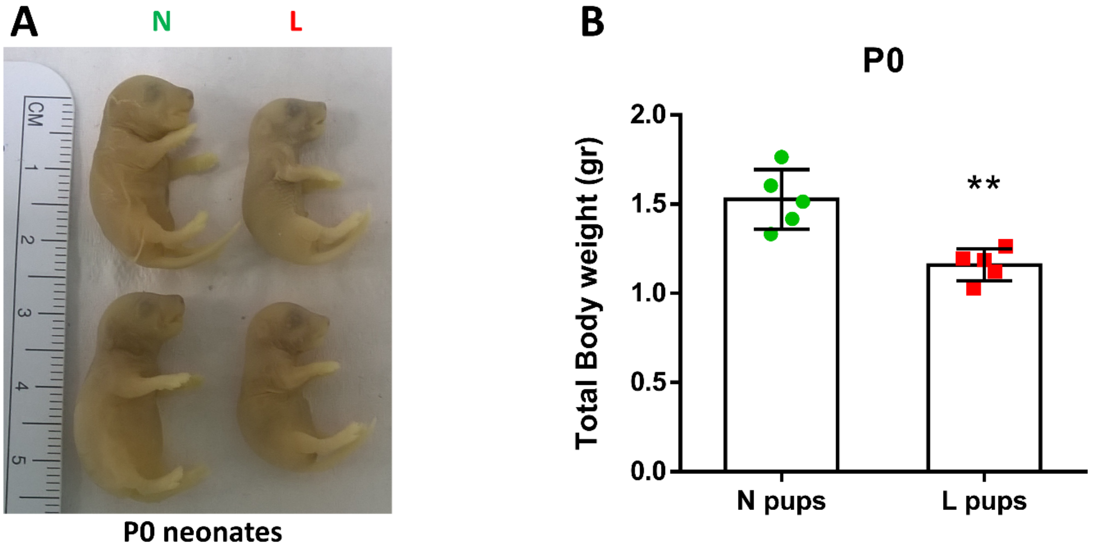


**Figure S1. Macroscopical differences between N and L pups.** Body size (A) and total body weight (B) of pups at birth (P0 neonates) born from dams on normal (N) and low (L) protein diet during gestation showed significant differences between the two groups. ***p*<0.01.

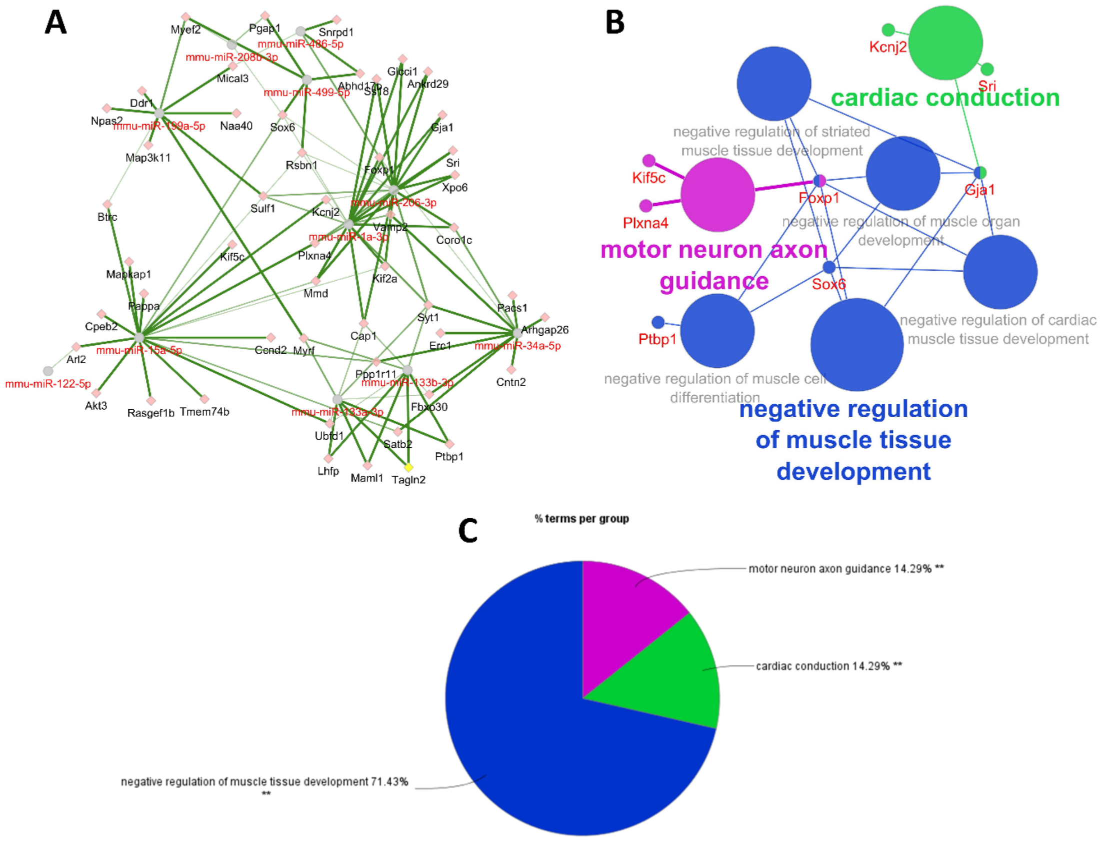

**Figure S2. Predicted gene target analysis for combined selected miRs and myomiRs.** Target analysis using Cytoscape showed that selected miRs (miR-15a, -34a, -199a and -122) and myomiRs have common predicted gene targets and can build an interactive network with several paths of molecular communication (A). KEGG analysis indicated that this miR set is implicated in musclx10associated biological pathways (B). The majority of genes participate in negative regulation of muscle tissue development and motor neuron axon guidance (C) by a statistically significant manner. $^{**}p<0.01$.

**Table S1. MiR primer list used for qPCR validation of small RNA-seq data.**

| Mature miR ID | miRbase Accession No | Sequence | miScript Primer Assay Catalog # |
|---|---|---|---|
| mmu-miR-199a-5p | MIMAT0000229 | CCCAGUGUUCAGACUACCUGUUC | MS00032529 |
| mmu-miR-122-5p | MIMAT0000246 | UGGAGUGUGACAAUGGUGUUUG | MS00001526 |
| mmu-miR-15a-5p | MIMAT0000526 | UAGCAGCACAUAAUGGUUUGUG | MS00001281 |
| mmu-miR-34a-5p | MIMAT0000542 | UGGCAGUGUCUUAGCUGGUUGU | MS00001428 |
| mmu-miR-206-3p | MIMAT0000239 | UGGAAUGUAAGGAAGUGUGUGG | MS00001632 |
| RNU6A-2 | ENSMUSG00000106147 | - | MS00033740 |

**Table S2. Differentially expressed miRs.** The list of 92 common miRs, as illustrated in Figure 2D, with significant DE between NN and NL groups and the corresponding log$_2$FC and FDR.

| miR | NN-NL log2FC | NN-NL FDR | NN-LN log2FC | NN-LN FDR | miR | NN-NL log2FC | NN-NL FDR | NN-LN log2FC | NN-LN FDR |
|---|---|---|---|---|---|---|---|---|---|
| mmu-miR-673-5p | -3.05763 | 1.07x10$^{-18}$ | 0.339637 | 1 | mmu-miR-483-5p | -2.245759 | 1.04x10$^{-6}$ | -0.288709 | 1 |
| mmu-miR-615-3p | -2.26461 | 1.52x10$^{-15}$ | -0.05959 | 1 | mmu-miR-409-3p | -1.687599 | 1.05x10$^{-6}$ | 0.041407 | 1 |
| mmu-miR-485-5p | -2.340456 | 2.35x10$^{-12}$ | -0.111724 | 1 | mmu-miR-143-5p | 2.875980 | 2.25x10$^{-6}$ | -0.056947 | 1 |
| mmu-miR-770-3p | -4.262491 | 4.04x10$^{-11}$ | 0.244386 | 1 | mmu-miR-378a-5p | 1.50422 | 2.37x10$^{-6}$ | -0.010584 | 1 |
| mmu-miR-152-3p | 1.313760 | 1.73x10$^{-10}$ | 0.071343 | 1 | mmu-miR-339-5p | 2.429160 | 3.51x10$^{-6}$ | 0.01036 | 1 |
| mmu-miR-673-3p | -2.336180 | 2.37x10$^{-10}$ | 0.038225 | 1 | mmu-miR-142a-3p | 4.123858 | 3.61x10$^{-6}$ | 1.071495 | 1 |
| mmu-miR-667-3p | -2.275383 | 2.37x10$^{-10}$ | 0.255797 | 1 | mmu-miR-6944-3p | -3.070585 | 4.18x10$^{-6}$ | -1.100795 | 1 |
| mmu-miR-744-5p | -2.118406 | 5.38x10$^{-10}$ | -0.004367 | 1 | mmu-miR-335-5p | 2.000673 | 4.42x10$^{-6}$ | -0.012486 | 1 |
| mmu-miR-145a-5p | 2.016469 | 5.38x10$^{-10}$ | -0.144154 | 1 | mmu-miR-30X105p | 2.262817 | 4.68x10$^{-6}$ | -0.128322 | 1 |
| mmu-miR-369-3p | 2.974593 | 5.43x10$^{-10}$ | 0.395163 | 1 | mmu-miR-133a-5p | 3.591677 | 5.33x10$^{-6}$ | 0.021891 | 1 |
| mmu-miR-21a-5p | 2.084967 | 2.67x10$^{-9}$ | 0.027165 | 1 | mmu-miR-361-5p | 1.480664 | 6.79x10$^{-6}$ | 0.297332 | 1 |
| mmu-miR-221-3p | 1.888911 | 3.38x10$^{-9}$ | 0.121640 | 1 | mmu-miR-708-3p | -2.042536 | 7.13x10$^{-6}$ | -0.300369 | 1 |
| mmu-miR-16-5p | 2.347114 | 7.63x10$^{-9}$ | -0.045761 | 1 | mmu-miR-129-5p | -2.515538 | 8.64x10$^{-6}$ | 0.337259 | 1 |
| mmu-miR-493-3p | -1.323967 | 9.31x10$^{-9}$ | -0.110136 | 1 | mmu-miR-15b-5p | 2.400766 | 9.57x10$^{-6}$ | -0.028085 | 1 |
| mmu-miR-126a-5p | 3.847861 | 9.31x10$^{-9}$ | -0.18413 | 1 | mmu-miR-101a-3p | 2.01101 | 1.01x10$^{-5}$ | 0.091404 | 1 |
| mmu-miR-340-5p | 2.210479 | 9.31x10$^{-9}$ | 0.173373 | 1 | mmu-miR-362-5p | 3.718623 | 1.04x10$^{-5}$ | -0.136591 | 1 |
| mmu-miR-1964-3p | -2.900966 | 1.88x10$^{-8}$ | -0.411296 | 1 | mmu-miR-425-5p | 2.380233 | 1.92x10$^{-5}$ | -0.280940 | 1 |
| mmu-miR-20a-5p | 3.009354 | 1.88x10$^{-8}$ | 0.090608 | 1 | mmu-miR-29c-3p | 5.156529 | 2.46x10$^{-5}$ | 0.367401 | 1 |
| mmu-miR-1981-5p | -3.684597 | 2.41x10$^{-8}$ | 0.175477 | 1 | mmu-miR-144-3p | 5.414024 | 2.69x10$^{-5}$ | -0.442355 | 1 |
| mmu-miR-666-5p | -2.921950 | 2.69x10$^{-8}$ | 0.041695 | 1 | mmu-miR-107-3p | 1.302001 | 3.52x10$^{-5}$ | 0.086966 | 1 |
| mmu-miR-206-3p | -2.308377 | 2.69x10$^{-8}$ | 0.100512 | 1 | mmu-miR-186-5p | 1.329084 | 4.03x10$^{-5}$ | -0.073228 | 1 |
| mmu-miR-128-3p | -2.752064 | 2.93x10$^{-8}$ | -0.374457 | 1 | mmu-miR-503-5p | 4.212161 | 5.48x10$^{-5}$ | 0.084991 | 1 |
| mmu-miR-19b-3p | 4.747869 | 3.30x10$^{-8}$ | 0.101924 | 1 | mmu-miR-450a-5p | 1.753405 | 5.72x10$^{-5}$ | -0.018198 | 1 |
| mmu-miR-199b-5p | 2.788858 | 3.74x10$^{-8}$ | -0.057749 | 1 | mmu-miR-362-3p | 4.104737 | 6.11x10$^{-5}$ | -0.001050 | 1 |
| mmu-miR-504-5p | -2.162741 | 4.15x10$^{-8}$ | -0.23838 | 1 | mmu-miR-142a-5p | 5.717220 | 6.95x10$^{-5}$ | 0.576811 | 1 |
| mmu-miR-671-3p | -2.396943 | 4.29x10$^{-8}$ | 0.078911 | 1 | mmu-miR-136-3p | 2.73910 | 8.23x10$^{-5}$ | 0.019391 | 1 |
| mmu-miR-543-3p | -1.933086 | 7.46x10$^{-8}$ | 0.253193 | 1 | mmu-miR-34c-5p | 2.442385 | 8.81x10$^{-5}$ | -0.087407 | 1 |
| mmu-miR-433-3p | -2.667109 | 7.91x10$^{-8}$ | -0.254429 | 1 | mmu-miR-3535 | -1.822816 | 9.78x10$^{-5}$ | -0.481787 | 1 |
| mmu-miR-365-3p | 2.226678 | 7.91x10$^{-8}$ | 0.144708 | 1 | mmu-miR-486a-3p | -2.643341 | 0.000111874 | -0.786577 | 1 |
| mmu-miR-151-3p | -2.855112 | 8.09x10$^{-8}$ | -0.041651 | 1 | mmu-miR-486b-3p | -2.643341 | 0.000111874 | -0.786577 | 1 |
| mmu-miR-708-5p | 3.065078 | 1.12x10$^{-7}$ | 0.523028 | 1 | mmu-miR-7688-5p | -3.33481 | 0.000152885 | -0.154922 | 1 |
| mmu-miR-199a-5p | 2.68809 | 1.67x10$^{-7}$ | 0.022378 | 1 | mmu-let-7c-2-3p | 1.730011 | 0.000165055 | -0.05096 | 1 |
| mmu-miR-1198-5p | -2.665912 | 1.94x10$^{-7}$ | 0.100403 | 1 | mmu-miR-320-3p | -1.568492 | 0.000169109 | 0.189259 | 1 |
| mmu-miR-195a-5p | 1.356708 | 3.17x10$^{-7}$ | -0.112575 | 1 | mmu-miR-376a-5p | 2.768539 | 0.00018953 | -0.090614 | 1 |
| mmu-miR-122-5p | -5.362835 | 3.87x10$^{-7}$ | -3.90744 | 0.04635 | mmu-miR-381-5p | 3.609241 | 0.000229858 | 0.074526 | 1 |
| mmu-miR-370-3p | -1.882551 | 3.87x10$^{-7}$ | 0.274937 | 1 | mmu-miR-34a-5p | 4.293383 | 0.00024698 | 0.091972 | 1 |
| mmu-miR-29a-3p | 2.664637 | 3.87x10$^{-7}$ | 0.119576 | 1 | mmu-miR-1943-5p | -2.075798 | 0.000289542 | -0.285117 | 1 |
| mmu-miR-133b-3p | 2.280328 | 4.00x10$^{-7}$ | -0.305938 | 1 | mmu-miR-139-3p | -2.026821 | 0.000295353 | 0.208809 | 1 |
| mmu-miR-93-5p | 2.196253 | 4.45x10$^{-7}$ | -0.068007 | 1 | mmu-miR-154-3p | 4.546951 | 0.000295353 | 0.554045 | 1 |
| mmu-miR-299a-5p | 2.581289 | 4.45x10$^{-7}$ | -0.198126 | 1 | mmu-let-7f-5p | -1.341164 | 0.000322693 | 0.069025 | 1 |
| mmu-miR-17-5p | 2.716892 | 4.68x10$^{-7}$ | 0.042478 | 1 | mmu-miR-144-5p | 2.183873 | 0.000398023 | -0.368364 | 1 |
| mmu-miR-374b-5p | 1.69365 | 5.73x10$^{-7}$ | 0.021264 | 1 | mmu-miR-185-5p | -1.386355 | 0.000425626 | 0.195121 | 1 |
| mmu-miR-133a-3p | 2.267177 | 5.73x10$^{-7}$ | -0.302002 | 1 | mmu-miR-1193-3p | 1.612390 | 0.000595312 | 0.336764 | 1 |
| mmu-let-7d-3p | -1.326561 | 6.32x10$^{-7}$ | 0.055221 | 1 | mmu-miR-690 | -2.267331 | 0.000635525 | -1.436904 | 1 |
| mmu-miR-541-5p | -1.784356 | 6.88x10$^{-7}$ | 0.046652 | 1 | mmu-miR-15a-5p | 3.812541 | 0.000793898 | -0.085752 | 1 |
| mmu-miR-434-5p | -2.036166 | 8.45x10$^{-7}$ | -0.077475 | 1 | mmu-miR-130b-5p | -1.635846 | 0.000840909 | 0.202233 | 1 |

**Table S3. GO analysis using IPA.** IPA-derived statistically significant GO which are associated with muscle and the list of predicted gene targets linked to each GO.

| Diseases or Functions Annotation | *p*-Value | Molecules | # Molecules |
|---|---|---|---|
| **Morphology of muscle** | **8.58x10$^{-12}$** | **IGFBP4,NFATC2,KLF15,MYCN,CCN2,USP18,PLD1,AGTR1,ROCK2,FTO,miR-133a-3p (and other miRNAs w/seed UUGGUCC),PPARGC1A,EDN1,HSD11B1,SPP1,BDNF,ESR1,CAV3,EGFR,ALS2,HOXA9,RB1CC1,HSPB8,APP,RCAN2,PTK2,PDPN,IGF1,ATP6V0C,IGF1R,PPARD,ZNF260,BAX,SOX4,NOTCH3,FMOD,UTRN,SLC6A6,ATE1,MYD88,LMO7,SRI,GATA6, TP53,DUSP1,PTPN11,ECE1,RAB1A,MET,CRK,HDAC4,TRIM63,CXADR,IL33,SIRT1,PDCD10,NFATC4,STK3,LRRC8A,TGFBR3,SLC9A1,CDK9,MBNL1,KLF2,RHOA,PAX7,SRF,PTGS2,SYNE1,MAML1,SERP1,MEF2A,CASP8** | **74** |
| **Morphology of muscle cells** | **2.76x10$^{-11}$** | **KLF15,NFATC2,CCN2,USP18,AGTR1,PLD1,ROCK2,FTO,PPARGC1A,EDN1,HSD11B1,SPP1,BDNF,CAV3,ALS2,HOXA9,EGFR,HSPB8,APP,PTK2,IGF1,ATP6V0C,PPARD,ZNF260,BAX,FMOD,UTRN,SLC6A6,ATE1,MYD88,LMO7,SRI,GATA6,TP53,DUSP1,PTPN11,ECE1,RAB1A,CXADR,TRIM63,SIRT1,IL33,NFATC4,STK3,TGFBR3,SLC9A1,CDK9,MBNL1,KLF2,RHOA,PAX7,PTGS2,SYNE1,MAML1,MEF2A** | **57** |
| **Differentiation of muscle** | **3.41x10$^{-11}$** | **MAPK11,JDP2,FOS,ZFP36L1,BDNF,CAV3,ZEB1,TMEM119,H3-3A/H3- B,AKIRIN1,PTK2,IGF1,RORA,CITED2,TNFRSF11B,PPARD,EZH2,RPTOR,CASP3,BAX,ARID1A,STK4,FGF6,NDN,RARA,EHMT2,FEM1C,GATA6,TP53,LDLR,PTPN11,QKI,FOXN2,MET,PLAGL1,DDX5,HDAC4,MEGF10,SIRT1,NFATC4,TCF3,SLC9A1,PDK4,MBNL1,RHOA,PAX7,CMTM5,SRF,SYNE1,MAML1,MEF2A,NACA** | **52** |
| **Proliferation of muscle cells** | **1.36x10$^{-10}$** | **MAPK11,IGFBP4,NFATC2,MYCN,PLD1,ROCK2,FOS,PPARGC1A,EDN1,LRP1,SPP1,BDNF,ESR1,F2,EGFR,TNFSF11,TCF4,TRIB1,PTK2,IGF1,CITED2,IGF1R,PPARD,BMPR2,VCAN,KLF11,NOTCH3,GJA1,FGF6,FGF1,RARA,MYD88,PAPPA,GATA6,TP53,DUSP1,PTPN11,RAB1A,MET,DNAJB6,HDAC4,CXADR,ID2,TGFBR3,SLC9A1,RAB5A,NFATC1,RHOA,PAX7,SRF,PTGS2,MAML1,TRIM32,NACA** | **55** |
| **Differentiation of muscle cells** | **3.98x10$^{-10}$** | **MAPK11,FGF6,NDN,RARA,GATA6,JDP2,TP53,FOS,LDLR,PTPN11,QKI,ZFP36L1,FOXN2,MET,PLAGL1,DDX5,HDAC4,MEGF10,BDNF,SIRT1,CAV3,NFATC4,TCF3,SLC9A1,ZEB1,TMEM119,H3-3A/H3-3B,AKIRIN1,MBNL1,PTK2,IGF1,RORA,CITED2,RHOA,PAX7,TNFRSF11B,PPARD,CMTM5,EZH2,SRF,SYNE1,MAML1,CASP3,BAX,ARID1A,MEF2A,STK4,NACA** | **48** |
| **Enlargement of muscle cells** | **3.93x10$^{-8}$** | **CCN2,USP18,AGTR1,PLD1,ROCK2,GATA6,TP53,miR-1-3p,miR-133a-3p,PPARGC1A,DUSP1,PTPN11,ECE1,EDN1,RAB1A,TRIM63,SIRT1,IL33,CAV3,NFATC4,STK3,EGFR,TGFBR3,SLC9A1,HSPB8,CDK9,PTK2,IGF1,RHOA,PPARD,PTGS2,ZNF260,MEF2A** | **33** |
| **Apoptosis of muscle cells** | **5.58x10$^{-8}$** | **MAPK11,NDN,BCL2L11,MCL1,BNIP3L,CNR1,PHB,ALDOA,AGTR1,PLD1,GATA6,TP53,PPARGC1A,PTPN11,EDN1,SPP1,CXADR,SIRT1,IL33,CAV3,PDCD4,TXNIP,ID2,HSPB8,APP,CDK9,FSTL1,IGF1,RHOA,IGF1R,TIMP3,PLD2,CASP3,DUSP6,STK4,NOTCH3,CASP8** | **37** |
| **Muscular hypertrophy** | **8.51x10$^{-8}$** | **CCN2,USP18,AGTR1,PLD1,ROCK2,GATA6,TP53,miR-1-3p, miR-133a-3p,PPARGC1A,DUSP1,PTPN11,ECE1,EDN1,RAB1A,TRIM63,SIRT1,IL33,CAV3,NFATC4,STK3,EGFR,TGFBR3,SLC9A1,HSPB8,CDK9,PTK2,IGF1,RHOA,PTGS2,ZNF260,MEF2A** | **32** |

**Table S4. Differentially expressed sncRNAs.** The list of 39 snRNAs, SNORA and SNORD with significant DE in NN against NL group and the corresponding $log_2$FC and FDR.

| Accession number | sncRNA | NN-NL log2FC | NN-NL FDR | NN-LN log2FC | NN-LN FDR |
|---|---|---|---|---|---|
| ENSMUSG00000065870 | RNU3a | -2.502290195 | $6.71x10^{-12}$ | -0.566464759 | 0.754672092 |
| ENSMUSG00000064390 | RNU73b | 2.279465326 | $9.33x10^{-11}$ | 0.121804384 | 1 |
| ENSMUSG00000077192 | SNORA17 | -1.60487376 | 0.001246879 | -1.09174037 | 0.348615031 |
| ENSMUSG00000064493 | SNORA28 | -2.667951294 | 9.18X1007 | -0.837325419 | 0.881241128 |
| ENSMUSG00000065939 | SNORA2b | 1.805342775 | 0.048394835 | -0.731328455 | 1 |
| ENSMUSG00000064602 | SNORA41 | -1.347154666 | 0.015406422 | -0.517879255 | 1 |
| ENSMUSG00000064949 | SNORA61 | -1.399403883 | 0.000631616 | -0.660500918 | 0.736260747 |
| ENSMUSG00000077563 | SNORA68 | 2.035928205 | 0.020547025 | -0.520216402 | 1 |
| ENSMUSG00000064387 | SNORA73a | -1.996602837 | 0.000916176 | -0.986284004 | 0.736260747 |
| ENSMUSG00000065353 | SNORA73b | -2.007307306 | 0.004480695 | -1.002878931 | 0.881241128 |
| ENSMUSG00000065649 | SNORA74a | -1.537201178 | 0.003695263 | -0.951670384 | 0.542405791 |
| ENSMUSG00000064837 | SNORA75 | -1.960454425 | $4.62x10^{-6}$ | -0.521341635 | 0.977667238 |
| ENSMUSG00000064513 | SNORA9 | 2.279923685 | $1.24x10^{-6}$ | 0.399654752 | 1 |
| ENSMUSG00000080486 | SNORD100 | 1.863174 | $3.77x10^{-7}$ | 0.295502 | 1 |
| ENSMUSG00000089317 | SNORD123 | 1.988553 | $2.84x10^{-20}$ | 0.368889 | 0.659504 |
| ENSMUSG00000094411 | SNORD16a | -1.32587 | $8.09x10^{-5}$ | -0.44689 | 0.899757 |
| ENSMUSG00000065094 | SNORD1a | 1.570603 | $7.33x10^{-5}$ | 0.755311 | 0.46057 |
| ENSMUSG00000077604 | SNORD1b | 1.80551 | $2.23x10^{-8}$ | 0.166508 | 1 |
| ENSMUSG00000065640 | SNORD1c | 2.295486 | $2.59x10^{-10}$ | 0.18701 | 1 |
| ENSMUSG00000088524 | SNORD2 | -1.5693 | 0.004447 | 0.013938 | 1 |
| ENSMUSG00000065087 | SNORD22 | -1.60426 | $7.20x10^{-9}$ | -0.12256 | 1 |
| ENSMUSG00000080478 | SNORD23 | -2.05375 | $2.53x10^{-5}$ | -1.23865 | 0.27793 |
| ENSMUSG00000065628 | SNORD33 | 2.105964 | $2.82x10^{-11}$ | 0.237865 | 1 |
| ENSMUSG00000064767 | SNORD35b | 1.757314 | $4.66x10^{-6}$ | 0.314847 | 1 |
| ENSMUSG00000064441 | SNORD37 | 1.325224 | $2.94x10^{-5}$ | 0.43 | 0.881241 |
| ENSMUSG00000065680 | SNORD38a | 2.646746 | $1.24x10^{-9}$ | 0.268185 | 1 |
| ENSMUSG00000064540 | SNORD42a | 1.453181 | $2.59x10^{-10}$ | 0.231646 | 1 |
| ENSMUSG00000064731 | SNORD45b | 1.799549 | $3.90x10^{-7}$ | 0.045265 | 1 |
| ENSMUSG00000065734 | SNORD49a | -1.39369 | $7.74x10^{-5}$ | 0.291939 | 1 |
| ENSMUSG00000077625 | SNORD4a | 2.032652 | $3.51x10^{-7}$ | 0.110771 | 1 |
| ENSMUSG00000065272 | SNORD57 | -1.49062 | 0.000899 | 0.180577 | 1 |
| ENSMUSG00000077345 | SNORD70 | 1.842033 | $3.90x10^{-9}$ | 0.122878 | 1 |
| ENSMUSG00000064984 | SNORD73a | 2.902044 | $1.48x10^{-8}$ | 0.143717 | 1 |
| ENSMUSG00000077220 | SNORD78 | 2.103305 | $9.31x10^{-6}$ | 0.22107 | 1 |
| ENSMUSG00000064823 | SNORD82 | -1.67109 | 0.000159 | 0.260947 | 1 |
| ENSMUSG00000077704 | SNORD89 | 2.119595 | $8.43x10^{-11}$ | 0.112696 | 1 |
| ENSMUSG00000077493 | SNORD91a | 1.310667 | $1.03x10^{-8}$ | 0.291039 | 0.922876 |
| ENSMUSG00000080469 | SNORD98 | 2.420401 | $1.73x10^{-13}$ | 0.101819 | 1 |
| ENSMUSG00000080615 | SNORD99 | -1.69718 | $4.84x10^{-5}$ | 0.445127 | 1 |
